# Collateral status, hyperglycemia, and functional outcome after acute ischemic stroke

**DOI:** 10.1186/s12883-022-02943-4

**Published:** 2022-11-04

**Authors:** Daniel F. Arteaga, Robin Ulep, Kevin K. Kumar, Andrew M. Southerland, Mark R. Conaway, James Faber, Max Wintermark, David Joyner, Vera Sharashidze, Karen Hirsch, Dan-Victor Giurgiutiu, Yousef Hannawi, Yasmin Aziz, Lori Shutter, Anita Visweswaran, Alana Williams, Kori Williams, Sonya Gunter, Heather M. Haughey, Askiel Bruno, Karen C. Johnston, Vishal N. Patel, SHINE Trial Investigators

**Affiliations:** 1 Department Neurology, St Thomas Rutherford Hospital, Murfreesboro, 1700 Medical Center Pkwy, Murfreesboro, TN 37129 USA; 2 Department of Neurology, Stanford University, Stanford, CA USA; 3 Department of Neurosurgery, Stanford University, Stanford, CA USA; 4 Department of Neurology, University of Virginia, Charlottesville, VA USA; 5 Department of Statistics, University of Virginia, Charlottesville, VA USA; 6 Department of Cell Biology and Physiology, University of North Carolina, Chapel Hill, NC USA; 7 Department of Radiology, Stanford University, Stanford, CA USA; 8 Department of Radiology, University of Virginia, Charlottesville, VA USA; 9 Department of Neurology, Emory University, Atlanta, GA USA; 10 Department of Neurology, Medical College of Georgia at Augusta University, Augusta, GA USA; 11 Department of Neurology, The Ohio State University, Columbus, OH USA; 12 Department of Neurology, University of Pittsburgh, Pittsburgh, PA USA; 13 Department of Critical Care Medicine, University of Pittsburgh, Pittsburgh, PA USA

**Keywords:** Intracranial collaterals, Hyperglycemia, Diabetes, Angiography, Ischemic stroke, Outcome

## Abstract

**Background:**

Mixed data exist regarding the association between hyperglycemia and functional outcome after acute ischemic stroke when accounting for the impact of leptomeningeal collateral flow. We sought to determine whether collateral status modifies the association between treatment group and functional outcome in a subset of patients with large vessel occlusion enrolled in the Stroke Hyperglycemia Insulin Network Effort (SHINE) trial.

**Methods:**

In this post-hoc analysis, we analyzed patients enrolled into the SHINE trial with anterior circulation large vessel occlusion who underwent imaging with CT angiography prior to glucose control treatment group assignment. The primary analysis assessed the degree to which collateral status modified the effect between treatment group and functional outcome as defined by the 90-day modified Rankin Scale score. Logistic regression was used to model the data, with adjustments made for thrombectomy status, age, post-perfusion thrombolysis in cerebral infarction (TICI) score, tissue plasminogen activator (tPA) use, and baseline National Institutes of Health Stroke Scale (NIHSS) score. Five SHINE trial centers contributed data for this analysis. Statistical significance was defined as a *p*-value < 0.05.

**Results:**

Among the 1151 patients in the SHINE trial, 57 with angiographic data were included in this sub-analysis, of whom 19 had poor collaterals and 38 had good collaterals. While collateral status had no effect (*p* = 0.855) on the association between glucose control treatment group and functional outcome, patients with good collaterals were more likely to have a favorable functional outcome (*p* = 0.001, OR 5.02; 95% CI 1.37–16.0).

**Conclusions:**

In a post-hoc analysis using a subset of patients with angiographic data enrolled in the SHINE trial, collateral status did not modify the association between glucose control treatment group and functional outcome. However, consistent with prior studies, there was a significant association between good collateral status and favorable outcome in patients with large vessel occlusion stroke.

**Trial registration:**

ClinicalTrials.gov Identifier is NCT01369069. Registration date is June 8, 2011.

**Supplementary Information:**

The online version contains supplementary material available at 10.1186/s12883-022-02943-4.

## Background

Acute or progressive steno-occlusive disease of the intracranial arterial network can trigger the induction of blood flow to the affected vascular territory via leptomeningeal collaterals. Enhanced collateral flow results in lumen enlargement of the supplying vasculature and subsequent dependence of affected parenchyma on collateral flow [1]. In patients and animal models with type two diabetes mellitus (T2DM), collateral perfusion is more susceptible to hemodynamic compromise relative to individuals without known T2DM [2, 3]. Mechanistically, this has been attributed to the negative effect of hyperglycemia on smooth muscle tone, conferring hypercoagulability and promoting collateral rarefaction, which collectively result in enhanced resistance within and downstream of the collateral network.

In the setting of acute ischemic stroke, the association between hyperglycemia and poor functional outcomes has been shown to be influenced by collateral status [4]. Several large retrospective analyses of patients with large vessel occlusions, including patients enrolled into the SWIFT, SWIFT PRIME, and STAR studies, found that the association between hyperglycemia (glucose level > 140 mg/dL) and poor functional outcome was diminished in those with poor collaterals [5–7]. However, older studies suggest that final infarct volume independent of collateral status in patients with hyperglycemia [8, 9].

The recently completed Stroke Hyperglycemia Insulin Network Effort (SHINE) trial demonstrated no significant difference in functional outcomes in hyperglycemic patients with acute ischemic stroke that received intensive compared to standard glucose control [10]. However, the SHINE study did not account for collateral status as a potential treatment effect modifier in either the primary or secondary endpoints. In the present study, we hypothesize that collateral status modifies the treatment effect of glucose control on functional outcome in hyperglycemic patients with large vessel occlusion acute ischemic stroke enrolled in the SHINE trial. Specifically, we hypothesized that intensive glycemic control will lead to improved functional outcomes relative to standard therapy in patients with good collaterals but not in patients with poor collaterals.

## Methods

### Patient selection

This post-hoc study was approved by the local institutional review board and ethics committee at each participating center. Sites which enrolled the highest number of large vessel occlusion patients were approached for inclusion into this analysis. Five of those sites agreed to participate and were included in the analysis. All enrolled patients provided written informed consent for the original trial.

Eligibility criteria for enrollment of hyperglycemic stroke patients in the SHINE trial is available from the primary results manuscript [7]. This study included additional imaging data that was completed as part of standard care but not originally collected for the SHINE trial. To create a standardized cohort for analysis, this sub-study only included patients with large vessel occlusions of the intracranial internal carotid artery or proximal middle cerebral artery on computed tomography angiography (CTA). The primary outcome was the degree of functional independence as assessed by the modified Rankin Scale (mRS) at 3 months.

### Collateral assessment

Assessment of collateral grade was performed using pretreatment single phase CTA. Collateral status was evaluated by two neuroradiologists (MW and DJ), each with 7 years or more of post-training neuroradiology experience. The evaluators were blinded to patient treatment groups and outcomes. Any disagreements after initial independent evaluation were resolved by discussion and consensus. Collaterals were stratified into good (2–3) or poor (0–1) categories based upon the grading system by Tan et al. [11].

### Statistical methods

The statistical analysis plan and primary outcome for this sub-study were determined prior to analysis. Categorical data were compared using χ ^2^ tests. To test the hypothesis that collateral status is an effect modifier between treatment groups, a multivariable logistic regression model was applied to the primary outcome. Adjustments to the model included the following predetermined variables: SHINE intervention group (categorical), collateral grade (categorical), thrombectomy status (categorical), age (continuous), post-perfusion thrombolysis in cerebral infarction (TICI) score (categorical), tissue plasminogen activator (tPA) use (categorical), and the National Institutes of Health Stroke Scale (NIHSS) score (categorical). Effect modification was determined by assessing the interaction between SHINE intervention group and collateral status. Statistical significance was defined as a *p*-value < 0.05.

## Results

### Study population baseline characteristics

Of the 1151 patients enrolled into the SHINE trial, 58 with angiographic data eligible for analysis. One patient was excluded because angiographic data could not be linked with the public SHINE database. Of the remaining 57 patients, 38 had good collaterals and 19 had poor collaterals (Table 1). Overall, the proportion of patients that underwent thrombectomy was similar between the good collateral group (66%) and the poor collateral group (58%). While 63% of patients in the poor collateral group received tPA, less than half (48%) of patients with good collaterals received tPA.

**Table 1:** Patient characteristics

Characteristics	Category	Poor Collaterals (***n*** = 19)	Good Collaterals (***n*** = 38)	Total	***P***-Value
		N (%)	N (%)	N (%)	
**Treatment Group**	Intensive	7 (37%)	24 (63%)	31 (54%)	0.060
	Standard	12 (63%)	14 (37%)	26 (46%)	
**Sex**	Male	11 (58%)	16 (42%)	27 (47%)	0.260
	Female	8 (42%)	22 (58%)	30 (53%)	
**Race**	Asian	2 (11%)	2 (5%)	4 (7%)	0.599
	Black	5 (26%)	7 (18%)	12 (21%)	
	Pacific Islander	0 (0%)	1 (3%)	1 (2%)	
	White	10 (53%)	26 (68%)	36 (63%)	
	Unknown	2 (11%)	2 (5%)	4 (7%)	
**Ethnicity**	Hispanic or Latino	3 (16%)	4 (11%)	7 (12%)	0.516
	Not Hispanic or Latino	15 (79%)	34 (89%)	49 (86%)	
	Unknown	1 (5%)	0 (0%)	1 (2%)	
**TPA**	No	7 (37%)	20 (52%)	27 (47%)	0.260
	Yes	12 (63%)	18 (48%)	30 (53%)	
**Prior Stroke**	No	17 (89%)	32 (84%)	49 (86%)	0.590
	Yes	2 (11%)	6 (16%)	8 (14%)	
**Diabetes**	No	4 (21%)	13 (34%)	17 (30%)	0.306
	Yes	15 (79%)	25 (66%)	40 (70%)	
**Atrial Fibrillation**	No	4 (31%)	9 (43%)	13 (38%)	0.481
	Yes	9 (69%)	21 (57%)	21 (62%)	
**Hypertension**	No	2 (11%)	6 (16%)	8 (14%)	0.590
	Yes	17 (89%)	32 (84%)	49 (86%)	
**Hyperlipidemia**	No	7 (37%)	14 (37%)	21 (37%)	1.00
	Yes	12 (63%)	24 (63%)	36 (63%)	
**Thrombectomy**	No	8 (42%)	13 (34%)	21 (37%)	0.560
	Yes	11 (58%)	25 (66%)	36 (63%)	
**TICI score**	Poor (0 – 2A)	0 (0%)	3 (12%)	3 (8%)	0.230
	Good (2B – 3)	11 (100%)	22 (88%)	33 (92%)	

*TICI* Thrombolysis in cerebral infarction

### Effect of collateral status

**Fig. 1:**
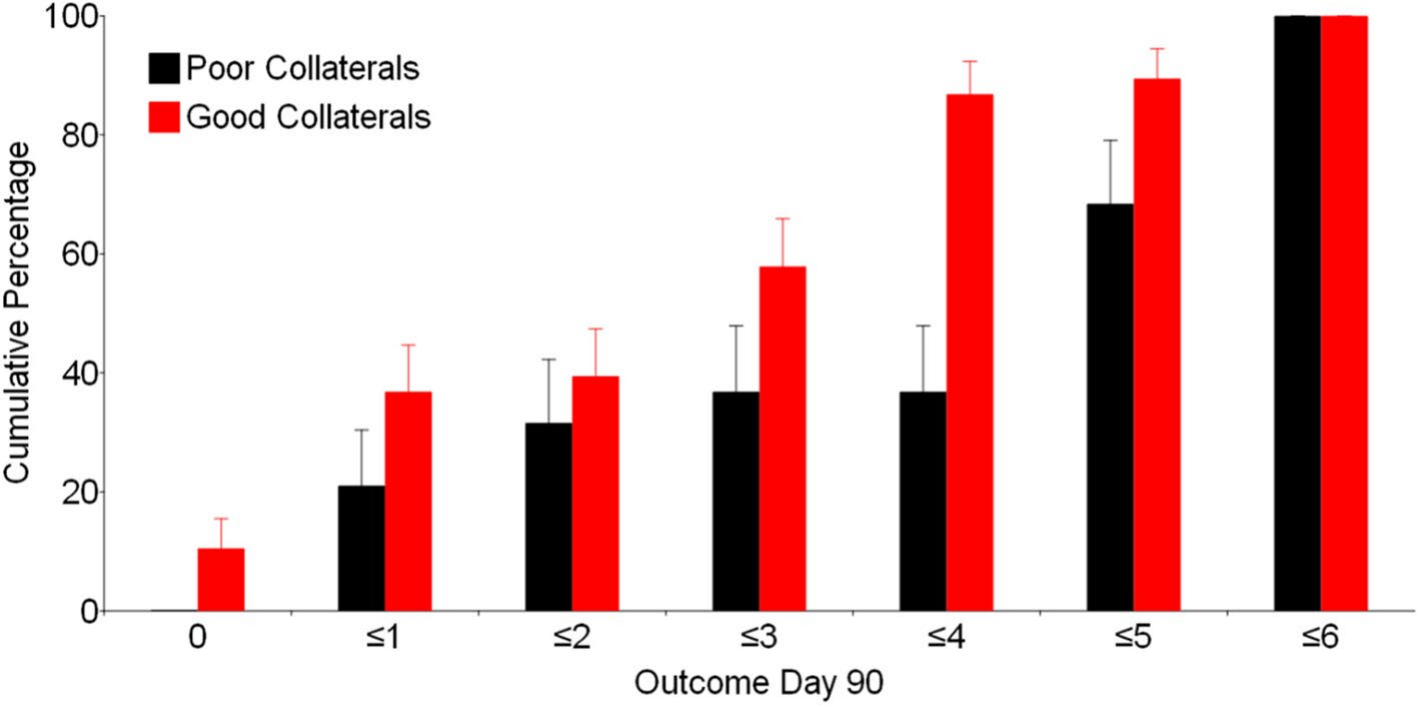
Association between collateral grade and 90-day mRS. mRS = modified Rankin Scale

The association between collateral status and functional outcome is presented in Fig. 1. Patients with good collaterals were more likely to have a favorable functional outcome (*p* = 0.001, OR 5.02; 95% CI 1.37–16.0), as defined by a mRS score of 0–2, when adjusted for age, sex, NIHSS, thrombectomy status, and TICI score. More detailed data, stratified across ordinal values in the mRS, can be seen in Supplemental Table 1. Collateral status did not modify the association between insulin treatment group and functional outcome (unadjusted *p*-value = 0.378; adjusted *p*-value = 0.855).

## Discussion

Our post-hoc analysis of the SHINE data reveals that the quality of collateralization did not significantly modify the association between functional outcomes and treatment with intensive compared to standard glucose control in patients with hyperglycemic acute ischemic stroke. However, the analysis confirmed that collateral status was directly associated with functional outcomes in patients with anterior circulation large vessel occlusion stroke, a finding consistent with previously published data [5].

Hyperglycemia is a common condition in patients presenting with acute ischemic stroke, with an incidence as high as 67% in this population [12, 13]. While several large retrospective analyses have demonstrated a negative association between high glucose levels and functional outcome [14, 15], the treatment of hyperglycemic stroke patients with intensive glucose control was not shown to improve functional outcomes in the SHINE trial [10]. In the present *post-hoc* study of the SHINE trial, we stratified patients by collateral status with the hypothesis that intensive glucose control would improve outcomes in patients with good collaterals. However, in our patient population, no association with collateral status was seen (*p*-value = 0.378, unadjusted). One major difference between our study and similar studies [5–7], which identified collateral status as a statistically significant effect modifier between glucose levels and functional outcome, was sample size.

It is important to note that diabetes was not considered as an independent variable in the present study as the vast majority (80%) of patients enrolled in the SHINE trial were found to have this condition. Furthermore, while the relationship between diabetes and leptomeningeal collateralization has been extensively explored [3, 16, 17], modifiable conditions in the acute setting, such as hyperglycemia, are poorly explored. From a pathophysiologic perspective, collaterals provide an alternative conduit to ischemic tissue, and a may provide a route through which glucose can potentiate direct neurotoxic effects in already compromised brain parenchyma, potentially amplifying the effects of reperfusion injury [7].

While collateral status ultimately did not modify the association between glucose control and functional outcomes in our sub-analysis, we did identify a skewed patient profile within the included SHINE population. Although the difference was not statistically significant (*p* = 0.06), it is notable that a higher percentage (63%) of patients in the intense glucose control arm of the trial were classified as having good collateral flow compared to those in the standard arm (37%). This finding raises possibility that baseline differences in collateralization between treatment arms may have impacted other outcome metrics in the initial analysis. In addition, while statistical significance was not achieved (*p* = 0.25), patients with poor collaterals received tPA at a higher rate (63%) compared to those with good collaterals (48%), suggesting that additional factors such as hemodynamic instability or medical comorbidities may have decreased thrombectomy candidacy and/or affected collateralization status.

The sub-analysis was limited by the relatively small number of patients that had angiographic data for inclusion. While we could not include all patients with angiographic data in the SHINE trial due to resource and technical limitations, we were able to select patients from the centers with the highest enrollment numbers. In addition, other cardiovascular risk factors such as hypertension, hyperlipidemia, and smoking history may have influenced the baseline level of collateralization after stroke and responsiveness to aggressive glucose management. However, due to limited power as a result of a smaller study size, we were unable to include these additional covariates. Given this limited power, it was decided a priori that only the primary endpoint of the SHINE trial would be considered when assessing the effect of collateral status. Additional exploratory analyses in future studies may help to identify the association of collateral status with secondary SHINE endpoints, including 90-day NIHSS score, 90-day Barthel Index score, and 90-day Stroke Specific Quality of Life Score. There is also the possibility of selection bias given that collateral status was not considered during patient randomization, and decisions to pursue thrombectomy were at the discretion of each site.

## Conclusions

In conclusion, we found that baseline collateral status did not modify the association between glucose control treatment group and functional outcome in patients with large vessel occlusion stroke. However, we did find a positive association between good collaterals and favorable outcome in this cohort, consistent with previously published research.

## Supplementary Information

**Additional file 1.**

## Data Availability

The datasets generated during and/or analyzed during the current study are available from the corresponding author on reasonable request.

## Abbreviations

SHINE: Stroke Hyperglycemia Insulin Network Effort
TICI: Thrombolysis In Cerebral Infarction
NIHSS: National Institutes of Health Stroke Scale
T2DM: Type Two Diabetes Mellitus
mRS: Modified Rankin Scale
tPA: Tissue Plasminogen Activator

## References

[1] Liebskind DS, Cotsonis GA, Saver JL, Lynn MJ, et al. Collateral circulation in symptomatic intracranial atherosclerosis. J Cereb Blood Flow Metab. 2011. 10.1038/2Fjcbfm.2010.224. 10.1038/jcbfm.2010.224 PMC3099635 21157476

[2] Moore SM, Zhang H, Maeda N, Doerschuk CM, Faber JE. Cardiovascular risk factors cause premature rarefaction of the collateral circulation and greater ischemic tissue injury. Angiogenesis. 2015. 10.1007/s10456-015-9465-6. 10.1007/s10456-015-9465-6 PMC4475464 25862671

[3] Menon BK, Smith EE, Coutts SB, Welsh DG, Faber JE, Goyal M, et al. Leptomeningeal collaterals are associated with modifiable metabolic risk factors. Ann Neurol. 2013. 10.1002/ana.23906. 10.1002/ana.23906 PMC3836863 23536377

[4] Prado R, Ginsberg MD, Dietrich WD, Watson BD, Busto R. Hyperglycemia increases infarct size in collaterally perfused but not end-arterial vascular territories. Cereb Blood Flow Metab. 1988. 10.1038/jcbfm.1988.48. 10.1038/jcbfm.1988.48 3343293

[5] Kim JT, Liebeskind DS, Jahan R, Menon BK, Goyal M, Nogueira RG, et al. Impact of hyperglycemia according to the collateral status on outcomes in mechanical thrombectomy. Stroke. 2018. 10.1161/STROKEAHA.118.022167. 10.1161/STROKEAHA.118.022167 30355207

[6] Genceviciute K, Goldlin MB, Kurmann CC, Mujanovic A, Meinel TR, Kaesmacher J, et al. Association of diabetes mellitus and admission glucose levels with outcome after endovascular therapy in acute ischaemic stroke in anterior circulation. Eur J Neurol. 2022. 10.1111/ene.15456. 10.1111/ene.15456 PMC9544025 35719010

[7] Feng W, Jiang B, Kanesan L, Zhao Y, Yan B. Higher admission fasting plasma glucose levels are associated with a poorer short-term neurologic outcome in acute ischemic stroke patients with good collateral circulation. Acta Diabet. 2018. 10.1007/s00592-018-1139-6. 10.1007/s00592-018-1139-6 29651557

[8] Shimoyama T, Shibazaki K, Kimura K, Uemura J, Shiromoto T, Watanabe M, et al. Admission hyperglycemia causes infarct volume expansion in patients with ICA or MCA occlusion: association of collateral grade on conventional angiography. Eur J Neurol. 2013. 10.1111/j.1468-1331.2012.03801.x. 10.1111/j.1468-1331.2012.03801.x 22747888

[9] Toni D, Manuela DM, Fiorelli M, Bastianello S, Camerlingo M, Sacchetti ML, et al. Influence of hyperglycemia on infarct size and clinical outcome of acute ischemic stroke patients with intracranial arterial occlusion. J Neurol Sci. 1994. 10.1016/0022-510x. 10.1016/0022-510x(94)90214-3 8064305

[10] Johnston KC, Bruno A, Pauls Q, Hall CE, Barrett KM, Barsan W, et al. Intensive vs standard treatment of hyperglycemia and functional outcome in patients with acute ischemic stroke: the SHINE randomized clinical trial. JAMA. 2019. 10.1001/jama.2019.9346. 10.1001/jama.2019.9346 PMC6652154 31334795

[11] Tan JC, Dillon WP, Liu S, Adler F, Smith WS, Wintermark M. Systematic comparison of perfusion-CT and CT-angiography in acute stroke patients. Ann Neurol. 2007. 10.1002/ana.21130. 10.1002/ana.21130 17431875

[12] Lindsberg PJ, Roine RO. Hyperglycemia in acute stroke. Stroke. 2004. 10.1161/01.STR.0000115297.92132.84. 10.1161/01.STR.0000115297.92132.84 14757880

[13] Zewde YZ, Mengesha AT, Gebreyes YF, Naess H. The frequency and impact of admission hyperglycemia on short term outcome of acute stroke patients admitted to Tikur Anbessa specialized hospital, Addis Ababa, Ethiopia: a cross-sectional study. BNC Neuro. 2019. 10.1186/s12883-019-1578-x. 10.1186/s12883-019-1578-x PMC6933642 31881850

[14] Kruyt N, Biessels GJ, Devries JH, Roos YB. Hyperglycemia in acute ischemic stroke: pathophysiology and clinical management. Nat Rev Neurol. 2010. 10.1038/nrneurol.2009.231. 10.1038/nrneurol.2009.231 20157308

[15] Ahmed N, Davalos A, Eriksson N, Ford GA, Glahn J, Hennerici M, et al. Association of admission blood glucose and outcome in patients treated with intravenous thrombolysis: results from the safe implementation of treatments in stroke international stroke thrombolysis register (SITS-ISTR). Arch Neurol. 2010. 10.1001/archneurol.2010.210. 10.1001/archneurol.2010.210 20837858

[16] Borggrefe J, Gluck B, Maus V, Onur O, Abdullayev N, Barnikol U, et al. Clinical outcome after mechanical thrombectomy in patients with diabetes with major ischemic stroke of the anterior circulation. World Neuros. 2018. 10.1016/j.wneu.2018.08.032. 10.1016/j.wneu.2018.08.032 30121406

[17] Lazzaro MA, Chen M, Christoforidis GA, Mohammad Y. The impact of diabetes on the extent of pial collateral in acute ischemic stroke patients. J Neuroi Surg. 2011. 10.1136/jnis/2010.004507. 10.1136/jnis.2010.004507 21990833

