# Supplementary material for: Collateral status, hyperglycemia, and functional outcome after acute ischemic stroke
Source: BMC Neurol. 2022 Nov 4;22:408. doi: 10.1186/s12883-022-02943-4 (PMC9635077; doi:10.1186/s12883-022-02943-4)
Supplement: Supplementary file 1 — Additional file 1. [file 12883_2022_2943_MOESM1_ESM.docx]

**Supplementary Table I. Categorical analysis between collateral grade and functional outcome.**

|  |  |  | Cumulative | |
| --- | --- | --- | --- | --- |
|  | Poor Collaterals  (n=19) | Good Collaterals  (n=38) | Poor Collaterals | Good Collaterals |
| MRS day 90 | N (%) | N (%) | N (%) | N (%) |
| 0 | 0 (0%) | 4 (11%) | 0 (0%) | 4 (11%) |
| 1 | 4 (21%) | 10 (26%) | 4 (21%) | 14 (37%) |
| 2 | 2 (11%) | 1 (3%) | 6 (32%) | 15 (39%) |
| 3 | 1 (5%) | 7 (18%) | 7 (37%) | 22 (58%) |
| 4 | 0 (0%) | 11 (29%) | 7 (37%) | 33 (87%) |
| 5 | 6 (32%) | 1 (3%) | 13 (68%) | 34 (89%) |
| 6 | 6 (32%) | 4 (11%) | 19 (100%) | 38 (100%) |

P = 0.001, chi-squared test.
